# Ultra-Sensitive Minute Mass Sensing Using a Microcantilever Virtually Coupled with a Virtual Cantilever

**DOI:** 10.3390/s20071823

**Published:** 2020-03-25

**Authors:** Yuki Kasai, Hiroshi Yabuno, Yasuyuki Yamamoto, Sohei Matsumoto

**Affiliations:** 1 Graduate School of System and Information Engineering, University of Tsukuba, Tsukuba, Ibaraki 305-8573, Japan; yuki.kasai.sy@alumni.tsukuba.ac.jp; 2 Fluid Property Standards Group, Research Institute for Engineering Measurement, National Metrology Institute of Japan (NMIJ), National Institute of Advanced Industrial Science and Technology (AIST), Tsukuba, Ibaraki 305-8563, Japan; 3 Research Center for Ubiquitous MEMS and Micro Engineering, National Institute of Advanced Industrial Science and Technology (AIST), Tsukuba, Ibaraki 305-8564, Japan

**Keywords:** minute mass sensor, mode localization, coupled resonator, MEMS sensor, RLC series circuit

## Abstract

Mass sensors based on the eigenmode shift of coupled cantilevers achieve much higher sensitivity than those based on the single cantilever’s eigenfrequency shift. In the former sensors, two identical cantilevers and a weak coupling stiffness between them are required to achieve high sensitivity. However, conventional coupled cantilevers cannot satisfy these requirements because of machining accuracy. To satisfy both requirements, a virtual coupling between a real macrocantilever and a virtual cantilever, whose dynamics was calculated using a digital computer, was proposed in our previous research. The sensitive mass sensing of mg-order masses was achieved. In the present work, for minute mass sensing, we replace the real macrocantilever with a real microcantilever. The calculation speed of a digital computer is not fast enough to calculate the virtual cantilever’s dynamics because the natural frequency of the microcantilver is much higher than that of the macrocantilever. Therefore, we use an analog circuit instead of a digital computer to achieve virtual coupling with the virtual cantilever. The proposed system enables us to tune the virtual cantilever’s parameters to satisfy both requirements for high sensitivity by changing the analog circuit parameters. We verified experimentally that the proposed system achieved high sensitivity for mass sensing of the order of nanograms.

## 1. Introduction

Recently, MEMS (Micro-Electro-Mechanical Systems) sensors using micro resonators have attracted much research attention [1,2,3,4,5,6]. Mass sensors based on the eigenmode shift of weakly coupled cantilevers [7] provide higher sensitivity in vacuum than those based on the eigenfrequency shift of a single cantilever [8,9,10]. We cannot measure biological samples alive in vacuum because they die. To realize mass measurements based on the eigenmode shift of weakly coupled cantilevers in viscosity environments, e.g., air and liquid, we applied the self-excited oscillation to weakly coupled cantilevers in our previous studies [11,12,13]. Various MEMS sensors for other measurement objects based on the eigenmode shift of weakly coupled cantilevers have also been developed [14,15,16]. In sensors based on the eigenmode shift of weakly coupled resonators, a pair of identical resonators with a weak coupling stiffness between them are required to achieve high sensitivity. However, these requirements are not sufficiently satisfied in practice because of limitations in machining accuracy. To achieve weaker coupling stiffness than the conventional coupled cantilevers using mechanical coupling like an overhang (for an example, see Figure 2 in Reference [7]), coupled resonators using electrical coupling have been proposed [17,18,19].

To achieve identical cantilevers in addition to weak coupling, we proposed the concept of virtual coupling between a real cantilever and a virtual cantilever in our previous research [20]. The dynamics of the virtual cantilever and the virtual coupling effect on the remaining real cantilever were calculated using a digital computer. The real cantilever was actuated in real time according to the virtual coupling effect. The mass sensor enabled us to produce identical cantilevers and a weak coupling stiffness between them, which are requirements for high sensitivity, because we could tune the physical parameters of the virtual cantilever and the virtual coupling effect arbitrarily in the digital scheme. We implemented a mass sensor using a macrocantilever, which had the eigenfrequency of 3.96 Hz, to clarify the validity of the proposed concept of virtual coupling between a real cantilever and a virtual cantilever. Our mass sensor using a real macrocantilever virtually coupled with a virtual cantilever demonstrated high sensitivity for the measurement of masses of the order of mg.

In our present study, for minute mass sensing, we replace the real macrocantilever used in our previous study with a real microcantilever. The natural frequency of the real microcantilever is much higher than that of the real macrocantilever used in our previous research. A higher calculation speed is necessary to calculate the dynamics of the virtual cantilever because the virtual cantilever must have the same high natural frequency as that of a real microcantilever. In this study, the virtual cantilever and the virtual coupling effect are implemented using an analog circuit instead of a digital computer. The analog circuit include an RLC series circuit to produce the dynamics of the virtual cantilever and the weak coupling. We confirmed experimentally that the proposed real microcantilever virtually coupled with a virtual cantilever satisfies both requirements for high sensitivity. We also verified experimentally that our proposed method resulted in higher sensitivity mass sensing of the order of nanograms compared with the conventional method based on the eigenfrequency shift of a single resonator.

## 2. Theory

### 2.1. Analytical Model of Weakly Coupled Cantilevers

**Figure 1:**
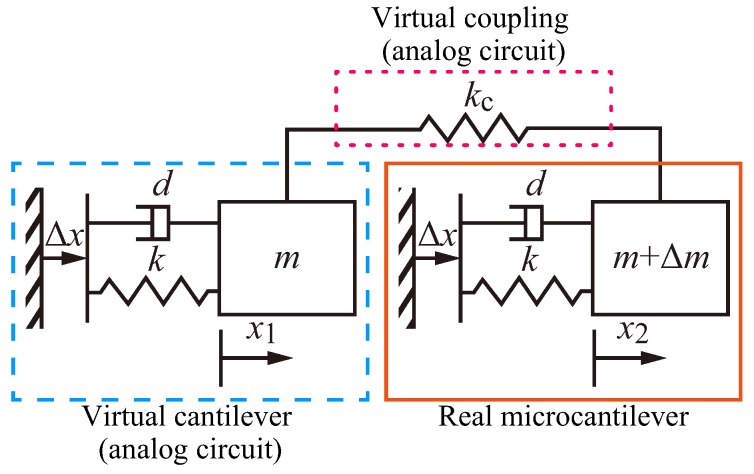
Discretized analytical model of the coupled real and virtual cantilevers. The left virtual cantilever enclosed by the dashed line and the virtual coupling part between cantilevers enclosed by the dotted line are implemented with an analog circuit. The right cantilever enclosed by the solid line is a real microcantilever.

We introduce a discretized analytical model of the coupled real and virtual cantilevers as Figure 1 based on the analytical model of coupled two real cantilevers in the previous studies [7,11], where *m* is the equivalent mass of the cantilevers, Δm is the mass to be measured, *k* is the equivalent stiffness of the cantilevers, $k_{c}$ is the equivalent coupling stiffness between the cantilevers, *d* is the viscous damping coefficient and Δx is the displacement at the support point that is actuated according to the feedback described below.

The relative displacements of the left and right cantilevers from the support point are denoted by $x_{1}$ and $x_{2}$, respectively. In the static equilibrium state, the displacements and the control input are $x_{1}=0$, $x_{2}=0$ and Δx=0, respectively. The left cantilever enclosed by the dashed line is implemented as a virtual cantilever, and the coupling part between cantilevers enclosed by the dotted line is implemented as a virtual coupling. The right cantilever enclosed by the solid line is implemented as a real microcantilever.

### 2.2. Mass Measurement Method Using Self-Excited Weakly Coupled Cantilevers

We first consider the coupled two real cantilevers used in the conventional setup [7,11] to obtain the actuation of the real cantilever at the support point. The equations of motion of the coupled cantilevers are expressed as follows:

(1)md2x1dt2+ddx1dt+kx1+kc(x1−x2)=md2Δxdt2(2)(m+Δm)d2x2dt2+ddx2dt+kx2+kc(x2−x1)=−(m+Δm)d2Δxdt2.

To measure directly the eigenmode without relying on the frequency response curve, we produce a self-excited oscillation as in our previous study [11,12]. We implement the feedback control to the coupled cantilevers using the displacement Δx at their supporting points. We can produce a self-excited oscillation using feedback as

(3) $$\Delta x=\alpha \int x_{2}dt,$$

where α is the feedback gain [11]. In this case, Equations (1) and (2) are expressed as follows:

(4)md2x1dt2+ddx1dt+mαdx2dt+kx1+kc(x1−x2)=0(5)(m+Δm)d2x2dt2+{d+(m+Δm)α}dx2dt+kx2+kc(x2−x1)=0.

The feedback can change the effect of damping. Another advantage of this method is that it allows mass sensing to be carried out even in viscosity environments because the feedback compensates for the viscosity. Actually, the feedback method enables us to measure minute masses in a high viscous liquid environment [13].

The first and second modal vectors of the coupled cantilevers, $p_{1},p_{2}$, can be expressed as

(6) $$p_{1}\approx \left[\begin{array}{c} 1-\frac{\delta }{2\kappa }\\ 1 \end{array}\right],p_{2}\approx \left[\begin{array}{c} -1-\frac{\delta }{2\kappa }\\ 1 \end{array}\right],$$

where the mass ratio and coupling ratio are denoted as δ=Δm/m and $\kappa \;=k_{c}/k$, respectively [7,11]. Generally, the displacements of coupled cantilevers, $x_{1}$ and $x_{2}$, are expressed using the first and second modal coordinates, $u_{1}$ and $u_{2}$, as follows:

(7) $$\left[\begin{array}{c} x_{1}\\ x_{2} \end{array}\right]=p_{1}u_{1}+p_{2}u_{2}.$$

However, under the self-excited oscillation realized by the feedback expressed as Equation (3), only the first mode is resonated and the second mode is attenuated immediately, i.e., $u_{2}=0$ [11]. Therefore, Equation (7) is rewritten as

(8) $$\left[\begin{array}{c} x_{1}\\ x_{2} \end{array}\right]=p_{1}u_{1}.$$

Thus, the amplitude ratio of coupled cantilevers can be regarded as the first eigenmode. In the case without a measured mass (δ=0), the amplitude ratio is 1:1; i.e., the amplitudes of the cantilevers are the same. When the measured mass is put on the real cantilever, the amplitude ratio is shifted greatly from 1:1 under the setting of a small κ, even if the measured mass is relatively very small compared with the mass of the cantilever; i.e., 0<δ≪1.

### 2.3. Implementation of a Virtual Coupling between a Real Microcantilever and a Virtual Cantilever

**Figure 2:**
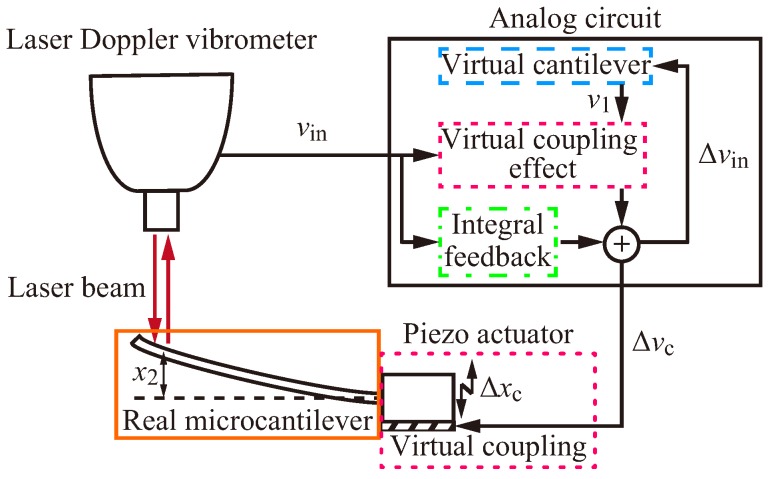
Schematic diagram of the experimental setup. The laser Doppler vibrometer measures the real microcantilever’s velocity. The output signal of the laser Doppler vibrometer $v_{\mathrm{in}}$ drives the analog circuit. The analog circuit calculates the displacement of the virtual cantilever $v_{1}$, the virtual coupling effect and the integral feedback for self-excited oscillation. The piezo actuator actuates the real microcantilever according to the output voltage of the circuit $\Delta v_{c}$ to realize the virtual coupling and the self-excited oscillation.

In this study, we implement the virtually coupled cantilevers using a real microcantilever and an analog circuit instead of a real macrocantilever and a digital computer [20]. The schematic diagram of the virtually coupled cantilevers is shown in Figure 2, where $v_{\mathrm{in}}$, $\Delta v_{c}$ and $\Delta x_{\mathrm{in}}$ are the input voltage to the analog circuit that represents the real microcantilever’s velocity, the output voltage that includes the virtual coupling effect and the voltage input to the RLC circuit mentioned below, respectively.

The laser Doppler vibrometer measures the real microcantilever’s velocity. The output signal of the laser Doppler vibrometer $v_{\mathrm{in}}$ drives the analog circuit. The analog circuit calculates the displacement of the virtual cantilever $v_{1}$, the virtual coupling effect and the integral feedback for self-excited oscillation. The piezo actuator actuates the real microcantilever according to the output voltage of the circuit $\Delta v_{c}$ to realize the virtual coupling and the self-excited oscillation.

**Figure 3:**
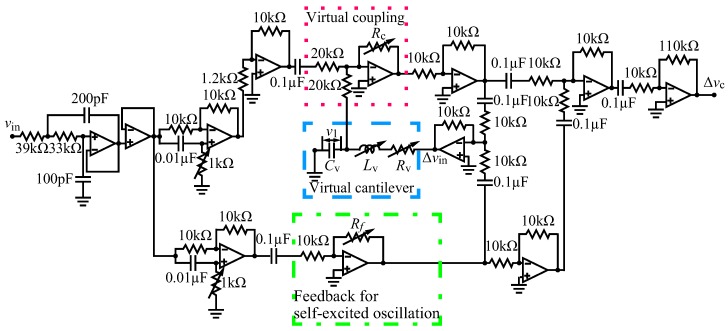
Analog circuit that implements the virtual cantilever and virtual coupling. The virtual cantilever’s dynamics are calculated by the RLC circuit enclosed by the dashed line. The resistance $R_{v}$ and inductance $L_{v}$ of the RLC circuit are tuned so that the virtual and real microcantilevers have the same eigenfrequency and damping ratio. The inter-terminal voltage of the capacitor in the RLC circuit is expressed as $v_{1}$. We can regard $v_{1}$ as a physical quantity that is proportional to the virtual cantilever’s displacement. The virtual coupling effect is calculated by the summing amplifier enclosed by the dotted line. The coupling stiffness between the real and virtual cantilever is tuned by the feedback resistance $R_{c}$. The feedback for self-excited oscillation is produced by the inverted amplifier enclosed by the chain line. The feedback gain for self-excited oscillation is tuned by the feedback resistance $R_{f}$.

The analog circuit used in the present research are shown in Figure 3.

The analog circuit includes an RLC series circuit, which has the same characteristics of oscillation as a cantilever. The virtual cantilever’s dynamics are expressed as the dynamics of the RLC circuit enclosed by the dashed line, where the resistance, inductance and capacitance are $R_{v},L_{v}$ and $C_{v}$, respectively. The virtual coupling effect is established by the summing amplifier enclosed by the dotted line in Figure 3. The virtual coupling effect is applied to the real cantilever by a piezo actuator to which the output voltage of the circuit $\Delta v_{c}$ is input. In this system, we make the physical parameters of the two cantilevers identical by tuning the impedance of the RLC circuit. The coupling ratio κ can be made extremely small by tuning the feedback resistance of the summing amplifier $R_{c}$ smaller as described below. To determine the actuation applied to the real microcantilever and the tuning of the analog circuit to achieve virtual coupling, we consider the circuit equation of the RLC circuit expressing the dynamics of the virtual cantilever and the equation of motion of the single cantilever as follows:

(9)Lvd2v1dt2+Rvdv1dt+1Cvv1=1CvΔvin(10)(m+Δm)d2x2dt2+ddx2dt+kx2=−(m+Δm)d2Δxcdt2.

The relationship between the output voltage of the analog circuit $\Delta v_{c}$ and the actuation by the piezo actuator $\Delta x_{c}$ is expressed as $\Delta x_{c}=d_{33}\Delta v_{c}$, where $d_{33}$ is the piezoelectric constant of the piezo actuator that moves the real microcantilever. The inter-terminal voltage of the capacitor in the RLC circuit is expressed as $v_{1}$. Since one of the real cantilevers is replaced with a virtual cantilever, Equation (1) changes into Equation (9). Additionally, because the coupling effect is achieved by virtual coupling, the fourth term on the left-hand side of Equation (2) disappears in Equation (10) and the virtual coupling effect is included in the right-hand side of Equation (10). We can regard $v_{1}$ as a physical quantity that is proportional to the virtual cantilever’s displacement $x_{1}$. We set the relationship between the voltage in the analog circuit and the cantilever’s displacement as $v_{i}=a_{x}x_{i}\left(i=1,2\right)$, where $a_{x}$ is a proportionality constant. In contrast, the real microcantilever’s displacement $x_{2}$ is obtained using a vibrometer. We set the resistance $R_{v}$ and inductance $L_{v}$ of the RLC circuit as $\sqrt{k/m}=1/\sqrt{L_{v}C_{v}}$ and $R_{v}=L_{v}d/m$ so that the virtual and real microcantilevers have the same eigenfrequency and damping ratio. In addition, we set $\Delta v_{\mathrm{in}}$ and $\Delta x_{c}$ as

(11)Δvin=−LvCvαaxdx2dt−kck(v1−axx2)(12)Δxc=∫∫αdx2dt+kcm(x2−v1ax)dtdt

so that the circuit equation of the RLC circuit, Equation (9), and the real cantilever’s equation of motion, Equation (10), are equivalent to the coupled two real cantilevers’ equations of motion, Equations (1) and (2), respectively. The first and second terms on the right-hand side of Equations (11) and (12) are the feedback to produce self-excited oscillation and to achieve virtual coupling, respectively. The feedback for self-excited oscillation is produced by the inverted amplifier enclosed by the chain line in Figure 3. The feedback gain α is tuned by the feedback resistance $R_{f}$. The feedback to generate virtual coupling is produced by the summing amplifier enclosed by the dotted line in Figure 3 with inverting input voltages of $v_{1}$ and $-a_{x}x_{2}$. Then, the coupling stiffness $k_{c}$ can be set small by tuning the feedback resistance $R_{c}$ small. This system enables us to satisfy the two stated requirements for high sensitivity mass sensing with identical cantilevers and weak coupling.

Our mass sensor completes the measurement of a mass within 50 ms. In this period, the temperatures of the microcantilever and the analog circuit are not changed so that the oscillation characteristics of the real and virtual cantilevers are stable. Once we adjust the oscillation characteristics of the real and virtual cantilevers by tuning the analog circuit, further tuning is not necessary.

## 3. Experiment

### 3.1. Experimental Setup

**Figure 4:**
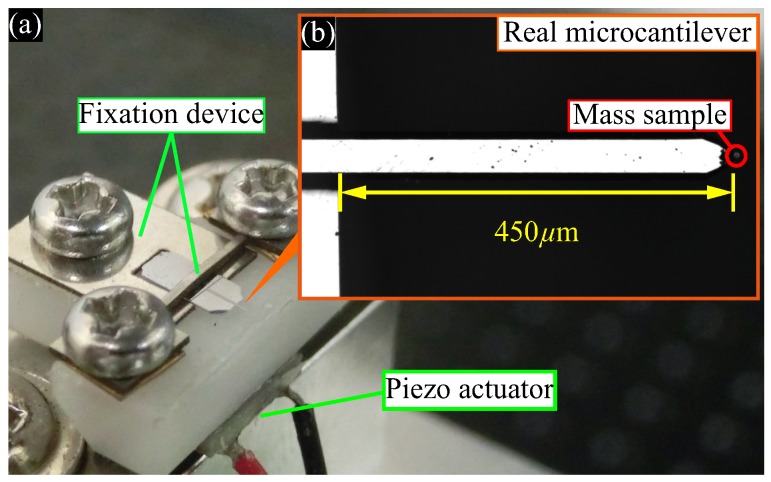
Experimental setup; (**a**) Piezo actuator and fixation device; (**b**) Real microcantilever. Minute particles with a mass of 17 ng as a mass sample on the real microcantilever which had dimensions of 450 μm × 55 μm × 4 μm and eigenfrequency of 24.7 kHz. The piezo actuator had a piezoelectric constant $d_{33}$ of 600 pm/V.

We demonstrated experimentally the identity of the real and virtual cantilevers and the weak coupling in the proposed system. Figure 4 shows the experimental setup of the piezo actuator and the real microcantilever.

We placed minute particles (Spherotech, Inc., Lake Forest, IL, USA: TP-60-5) with a mass of 17 ng as a mass sample on the real microcantilever shown in Figure 4b (Hitachi High-Technologies Corporation, Tokyo, Japan: SI-DF3) which had dimensions of 450 μm × 55 μm × 4 μm and eigenfrequency of 24.7 kHz. The laser Doppler vibrometer (Polytec GmbH, Waldbronn, Germany: MSA-500) measured the real microcantilever’s velocity. The piezo actuator shown in Figure 4a (FUJI CERAMICS Corp., Fujinomiya-shi, Shizuoka-ken, Japan: Z1T5×5S-SYXN (C-82)), which had a piezoelectric constant $d_{33}$ of 600 pm/V, actuated the real microcantilever with feedback actuation $\Delta x_{c}$, generating the feedback for self-excited oscillation and the virtual coupling effect.

### 3.2. Experimental Results

#### 3.2.1. Identity of Real and Virtual Cantilevers

**Figure 5:**
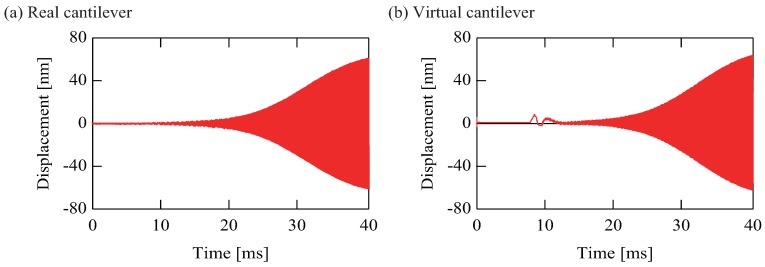
Time histories of self-excited virtually coupled cantilevers without mass samples; (**a**) Real microcantilever; (**b**) Virtual cantilever. The growing amplitudes of both the self-excited real and virtual cantilevers are nearly equal at each time. This result shows the physical parameters of the virtual cantilever can be made almost identical with those of the real microcantilever by tuning the impedance of the RLC circuit.

Figure 5 shows the time histories of self-excited virtually coupled cantilevers without mass samples; i.e., mass ratio δ=0, where (a) and (b) are for the real microcantilever and virtual cantilever, respectively. These cantilevers are excited only with the natural frequency of the first mode by the feedback expressed as Equation (3). The amplitudes of the self-excited cantilevers grow and reach the maximum measurement range of the vibrometer because we did not apply any amplitude control methods to the cantilevers. Such a saturation degrades the sensitivity and accuracy of the proposed mass sensing because the saturation effect is not taken into account in the measurement theory. To prevent the saturation effect, we measured the amplitudes of coupled cantilevers in transient state before they were saturated because the amplitude ratio in the transient state expresses the eigenmode. However, as mentioned in Section 3.2.3, we must use a further method to increase the measurement accuracy.

The growing amplitude of both the self-excited real and virtual cantilevers, which are obtained from the relationship between the voltage and the displacement of the cantilever as described previously; i.e., $v_{i}=a_{x}x_{i}\left(i=1,2\right)$, are nearly equal at each time. For example, at 31 ms, the virtual cantilever’s amplitude was 29.3 nm when the real microcantilever’s amplitude became 32.2 nm. This result shows the physical parameters of the virtual cantilever can be made almost identical with those of the real microcantilever by tuning the impedance of the RLC circuit.

#### 3.2.2. Coupling Ratio

Next, we examined the weak stiffness of the virtual coupling; i.e., the coupling ratio κ.

**Figure 6:**
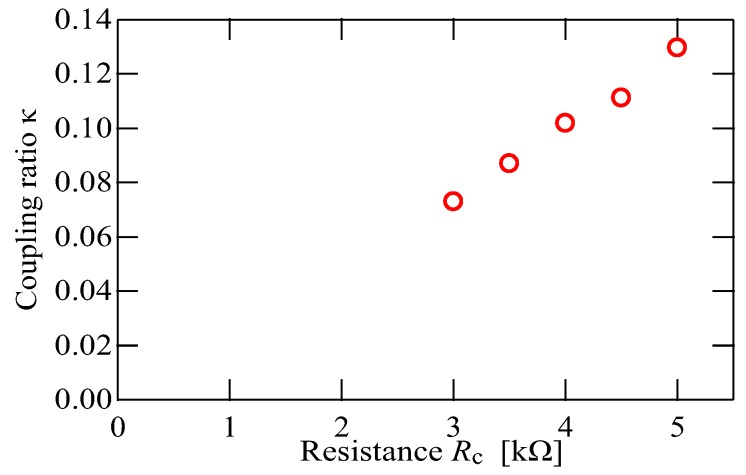
Relationship between the experimentally measured coupling ratio κ and the resistance $R_{c}$ used to tune the nominal coupling ratio. The experimentally measured coupling ratio was proportional to the resistance $R_{c}$. Setting the resistance $R_{c}$ smaller achieves a smaller coupling ratio, and a smaller coupling ratio κ will produce a higher sensitivity.

Figure 6 shows the relationship between the experimentally measured coupling ratio and the variable resistance tuned for a weak coupling. The experimentally measured coupling ratio was proportional to the resistance $R_{c}$. This result accords with the theoretical circuit design mentioned before. Setting the resistance smaller will give a smaller coupling ratio, and according to Equation (6), a smaller coupling ratio κ will produce a higher sensitivity. Thus, our virtually coupled cantilevers can achieve sensitive measurement of minute masses.

#### 3.2.3. Mass Measurement

**Figure 7:**
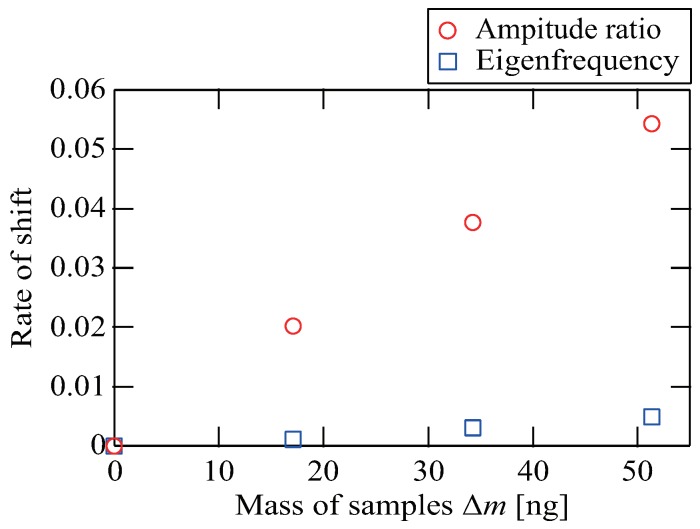
Minute mass measurement result. The horizontal and vertical axes represent the mass of the samples and the rate of shift, respectively. The red circles and the blue squares represent the average rate of shift of the amplitude ratio of the virtually coupled cantilevers and the average rate of shift of the eigenfrequency of the single cantilever, respectively. We set the virtual coupling ratio κ at 0.073 in this experiment. The amplitude ratio of the virtually coupled cantilevers changed in proportion to the added mass. The sensitivity of the virtually coupled cantilevers was over 10 times higher than the conventional method based on the single cantilever’s eigenfrequency shift.

Finally, we performed the measurement of minute mass samples in air and compared the sensitivity of the prototype measurement system with that of the conventional method based on the eigenfrequency shift of a single cantilever. In the previous research [7], the mass sensing of the order of picogram order in vacuum was achieved. On the other hand, we realized mass sensing of nanogram order in air using self-excited oscillation. We prepared three kinds of mass samples using a minute particle with a mass of 17 ng. The mass samples A, B and C consisted of one, two, and three particles, respectively. We measured the shift in the amplitude ratio of the virtually coupled cantilevers and the eigenfrequency shift of the single cantilever five times when each mass samples were placed on the real cantilevers. Then, we set the virtual coupling ratio κ at 0.073. Figure 7 shows the results of the minute mass measurements, where the horizontal and vertical axes represent the mass of the samples and the rate of shift, respectively. The red circles and the blue squares represent the average rate of shift of the amplitude ratio of the virtually coupled cantilevers and the average rate of shift of the eigenfrequency of the single cantilever, respectively.

The amplitude ratio of the virtually coupled cantilevers changed in proportion to the added mass. This result agrees with the theoretical analysis expressed by Equation (6). The mass sample C with a mass of 51 ng changed the amplitude ratio by 5.4%. The sensitivity of our proposed method was 1.06/μg. With the conventional method, the mass sample C changed the eigenfrequency by only 0.49%, giving a sensitivity of 0.0985/μg. Therefore, our proposed virtually coupled cantilevers achieved a sensitivity over 10 times higher than the conventional method based on the single cantilever’s eigenfrequency shift.

Next, we consider the comparison from the viewpoint of the accuracy using the relative errors of measurement results, where the average value is assumed as the true value. We calculated the relative errors of the experimentally measured amplitude ratio and eigenfrequency when each of the three kinds of mass sample is on cantilever. We obtained 15 relative errors by five measurements for each mass sample. The averages of the relative errors of the amplitude ratio measurement and the frequency measurement are $3.57\times 10^{-3}$ and $2.74\times 10^{-4}$, respectively. Therefore, the measurement accuracy of the amplitude ratio is about 13 times worse than that of the frequency. One possible reason is that the mode of the cantilevers is estimated from the amplitude ratio in the transient state, which can be easily influenced by disturbances while sensing. Therefore, this issue must be solved to increase the measurement accuracy of amplitude. As a detection method for the amplitude, which does not use the oscillation in transient state or depend on the saturation effect mentioned in Section 3.2.1, we can employ the additional application of nonlinear feedback as proposed in our previous research [13]. The nonlinear velocity feedback produces the self-excited oscillation with the small constant steady state amplitude, i.e., a limit cycle with a small radius, in the coupled cantilevers and enables us to measure the mode in the steady state. This method can be more robust against the disturbance and not affected by the saturation. The application remains as a future work.

## 4. Conclusions

In conclusion, using the concept of virtual coupling between a real cantilever and a virtual cantilever as the basis for a highly sensitive measurement method, we successfully implemented a practical minute mass measurement system using an analog circuit. We verified experimentally that our proposed measurement system realizes a pair of identical cantilevers and weak coupling stiffness between them, which are requirements for high sensitivity. In mass sensing of the order of nanograms, our proposed minute mass sensor achieved a sensitivity 10 times higher than the conventional method using the eigenfrequency shift of a single cantilever.
